# Linking Chemical Composition to Thermal Decomposition of Lignocellulosic Biomass and Its Structural Fractions: Insights from Eucalyptus and Sugarcane Bagasse

**DOI:** 10.1021/acsomega.6c04390

**Published:** 2026-09-27

**Authors:** Natália R. de Carvalho, Anna J. B. Correia, Gabriela T. Nakashima, Ana Larissa S. Hansted, Ariane A. F. Pires, Tamaki Kugimiya, Hiroyuki Yamamoto, Fábio M. Yamaji

**Affiliations:** † Federal University of São Carlos, Department of Environmental Sciences, João Leme dos Santos Highway, Km 110 - SP-264, Itinga Neighborhood, Sorocaba, São Paulo 18052-780, Brazil; ‡ Brazilian Aluminium Company, Alumínio, São Paulo 18125-000, Brazil; § Department of Fisheries, Forestry, and Agriculture, Government of Newfoundland and Labrador, St. John’s, NL A1B 4J6, Canada; ∥ Graduate School of Bio-agricultural Sciences, Nagoya University, Chikusa, Nagoya 464-8601, Japan

## Abstract

Lignocellulosic biomass is considered a promising alternative to fossil fuels. Due to its complex composition, understanding its thermal behavior is essential for improving the energy efficiency of thermochemical conversion processes. Thermogravimetric analysis has been widely used to investigate biomass thermal behavior; however, available studies are generally fragmented across different materials and experimental conditions, limiting direct comparisons. Therefore, this study provides a systematic comparison of eucalyptus wood and sugarcane bagasse, together with their structural fractions, under identical thermogravimetric conditions. This study investigates the thermal decomposition behavior of eucalyptus wood chips and sugarcane bagasse, as well as their structural components—cellulose, hemicellulose, and lignin—to better understand biomass pyrolysis processes. Proximate and chemical analyses were conducted to characterize the biomass and isolate its structural fractions, followed by thermogravimetric (TG/DTG) analysis. In addition, DTG curve deconvolution was performed to quantitatively evaluate overlapping degradation events. In general, eucalyptus and sugarcane bagasse exhibited similar TG and DTG profiles under equivalent conditions, including the in natura biomass and samples after sequential removal of extractives, hemicellulose (holocellulose), and α-cellulose. However, a gradual disappearance of the shoulder peak (*T*_sh_) preceding the maximum decomposition temperature, together with a narrowing of the main DTG peak, was observed after the sequential removal of structural components, indicating the influence of extractives and hemicellulose on thermal decomposition. Deconvolution analysis quantitatively confirmed the contribution of these overlapping thermal events. These results establish a direct relationship between chemical composition and thermal behavior under standardized conditions. The comparative evaluation of biomass and its structural fractions, complemented by DTG curve deconvolution, provides quantitative information that improves the interpretation of thermal degradation events and supports the development of thermochemical conversion technologies for bioenergy applications.

## Graphical abstract

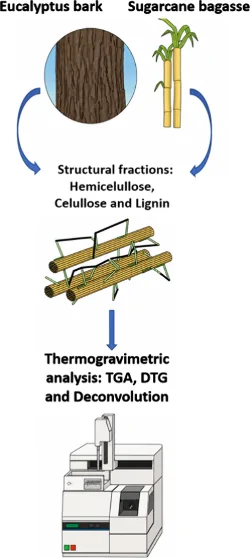

## 1 Introduction

Biomass is a renewable energy source that is widely available and used worldwide, with the aim of reducing the use of fossil fuels and, consequently, mitigating climate change and promoting energy independence.^1^ The use of lignocellulosic biomass for energy generation, broadly and disregarding some aspects, has a zero-carbon balance, since the carbon released during burning is captured from the atmosphere during the photosynthesis process carried out by plants.^2^

To ensure energy efficiency, it is necessary to understand the thermal degradation behavior of biomass during pyrolysis. Through thermogravimetric analysis of biomass, it is possible to study the kinetic parameters and understand the thermal degradation processes of biomass.^3^ Thermogravimetric analysis is a fast and efficient analytical technique that allows one, with a small sample quantity, to plot mass-loss curves as a function of time or temperature, under inert or oxidizing atmospheres.^4,5^ By using thermogravimetry, it is possible to increase energy efficiency by identifying the temperature range suitable for pyrolysis processes for each type of biomass, the existing endothermic and exothermic reaction phases, and the mass loss associated with fuel quality.^6^ Biomass is mainly composed of cellulose (40% to 50%), lignin (10% to 40%), and hemicelluloses (20% to 40%).^7–12^ Since the temperature ranges of hemicellulose and cellulose overlap and the decomposition of lignin encompasses a wide temperature range,^8^ the precise definition of where the decomposition of each biomass component begins and ends is impaired. Therefore, the isolated thermal study of these compounds would facilitate the identification of the decomposition ranges of each component of lignocellulosic biomass and how they interfere with the thermogravimetric curve.

Although thermogravimetric analyses of lignocellulosic biomass have been widely reported, the available studies remain fragmented, focusing on individual biomasses, isolated structural components, reference compounds, or specific pretreatment processes. Based on the literature reviewed, no studies were identified that systematically compare eucalyptus wood and sugarcane bagasse under identical thermogravimetric conditions while simultaneously evaluating whole biomass, extractive-free biomass, holocellulose, α-cellulose, isolated lignin fractions, and pure reference compounds within a single experimental framework. Consequently, direct correlations between the sequential removal of structural constituents and the corresponding changes in TG/DTG profiles remain limited.

Therefore, the objective of the study was to thermally analyze the sugarcane bagasse and eucalyptus biomass, as well as the main biomass components—cellulose, hemicellulose, and lignin—in order to identify the degradation patterns of the components observed in the biomass thermogravimetric curves. This approach provides a more comprehensive interpretation of the relationship between biomass chemical composition and thermal degradation behavior.

## 2 Experimental Section

To analyze the thermal behavior of biomass and its compounds, materials considered as standards for biomass components, in natura biomass, and materials resulting from chemical analysis processes of biomass were analyzed. Cotton and microcrystalline cellulose were used as standards to represent cellulose. Very pure xylose was used for hemicellulose, and kraft and organosolv lignins were used to represent lignin. The biomass feedstocks analyzed were sugarcane bagasse (from a sugarcane mill) and eucalyptus residue (from a wood panel industry). The biomass were prepared for chemical analysis according to the standards. Samples of each stage were taken to perform thermogravimetry analysis, obtaining the biomass in the following conditions: in natura (all of the components in their original form); biomass after the extraction process (biomass without extractives); holocellulose (composed of cellulose and hemicellulose); α-cellulose (does not contain hemicelluloses and lignin); and insoluble Klason lignin. Each procedure is detailed below.

The sugarcane bagasse and eucalyptus chip samples were previously crushed in a Willey MA-340 type mill and dried in an oven at a temperature of 103 ± 2 °C until the sample weight stabilized. The dry base moisture of the biomass was determined on a moisture balance.

The proximate analysis was performed in duplicate. The crucibles were previously calcined to avoid contamination in the samples. 1.5 g of material was weighed for each repetition in a crucible with a lid. The crucibles were placed in a muffle furnace at 900 °C for 10 min, 3 min with the muffle door open and 7 min with the door closed. Afterward, the crucibles were conditioned in a desiccator for 30 min and weighed.^13^ The calculation of volatile contents was done according to eq 1:

1 $$TV\;\left(\%\right)=\frac{m_{i}-m_{f}}{m_{i}}\times 100$$

where TV is the volatile content (%), *m*_i_ is the initial mass (g), and *m*_f_ is the final mass (g).

Then, the ash analysis was performed. The crucibles were placed in the muffle furnace at 600 °C for 6 h (until a constant weight was reached). The ash content was calculated according to eq 2:

2 $$TC\;\left(\%\right)=\frac{m_{2}-m_{1}}{m}\times 100$$

where TC is the ash content (%), *m* is the mass of the sample free of moisture (g), *m*_1_ is the mass of the crucible (g), and *m*_2_ is the mass of the crucible plus the residue after the period in the muffle furnace (g).

The fixed carbon content (TCF) was determined according to eq 3:

3 TCF=100−(TV+TC)

where TCF is the fixed carbon content (%), TC is the ash content (%), and TV is the volatile content (%).

Then, chemical analysis was carried out, which included extractives, Klason lignin, holocellulose, α-cellulose, and ash.

The extractive content was determined via Soxhlet extraction using cyclohexane/ethanol as well as ethanol and hot water according to TAPPI T 204 cm-97,^14^ with the flow adjusted to occur at least 24 cycles in a period of 4 to 5 h. After this period, the samples were dried in an oven at 60 °C for 24 h and weighed to determine the total extractive content.

The insoluble Klason lignin content was determined based on ASTM D1106-96.^15^ The analysis was performed in duplicate; for each repetition, 1 g of dry material without extractives was weighed and conditioned in a beaker with 15 mL of aqueous sulfuric acid solution (H_2_SO_4_) at a temperature of 10 to 15 °C. The mixture was macerated and kept in an ice bath at 2 °C. Then, the mixture with the completely dispersed material was sealed and kept under stirring at 20 °C for 2 h. After this period, 200 mL of distilled water was added, and the mixture from the beaker was transferred to a 500 mL Erlenmeyer flask. The volume was completed with distilled water until it reached the 300 mL mark, obtaining a diluted 3% H_2_SO_4_ solution that was boiled for 4 h, maintaining a constant volume. The ice bath was repeated for 30 min, and the solution was vacuum filtered, taking care to wash the solution with distilled water until it reached neutral pH. The filter paper was dried in an oven at 60 °C for 24 h and weighed on an analytical balance to calculate the Klason lignin content.

The holocellulose content was determined based on ASTM D1104-56.^16^ The analysis was performed in duplicate for each treatment. For each repetition, 3 g of material without extractives was weighed and stored in a 500 mL Erlenmeyer flask containing 120 mL of distilled water, 2.5 g of 80% sodium chlorite, and 1 mL of glacial acetic acid. The container was covered and heated in a water bath at 70 °C under constant stirring. Two additions of 2.5 g of sodium chlorite and 1 mL of glacial acetic acid were made after 1 and 2 h of analysis. After the last addition, the container was kept heated and stirred for another 3 h, totaling 5 h of experiment time. Then, an ice bath was created for 30 min, and the solution was vacuum filtered, taking care to wash the solution with distilled water until it reached neutral pH. The filter paper was dried in an oven at 60 °C for 24 h and weighed on an analytical balance to calculate the holocellulose content.

The α-cellulose content was determined based on ASTM D1103-60.^17^ The analysis was performed in duplicate for each treatment. For each repetition, 1 g of holocellulose was weighed, and 10 mL of sodium hydroxide (NaOH) solution at a concentration of 17.5% was added. The mixture was left to stand for 2 min and macerated for 8 min. After that, another 10 mL of the 17.5% NaOH solution was added. After another 20 min of standing, 40 mL of distilled water was added, and vacuum filtration was performed, taking care to wash the solution with distilled water until it reached neutral pH. The filter paper was dried in an oven at 60 °C for 18 h and weighed on an analytical balance to calculate the α-cellulose content.

Thermogravimetric analyses (TGA) were performed using a Pyris 1 TGA Thermogravimetric Analyzer (PerkinElmer), in the temperature range of 30 to 950 °C at a heating rate of 20 °C min^–1^, under a continuous flow of high-purity nitrogen gas (N_2_) 99.999% at 20 mL min^–1^, with a platinum crucible.^13,18,19^ The TGA measurements were performed in duplicate for each sample, and a representative curve was selected for graphical presentation. For samples exhibiting complex DTG profiles with multiple overlapping peaks, a third measurement was performed to confirm the reproducibility of the observed thermal behavior. Subsequently, the test data were used to generate the thermogravimetric (TG) curves and the derivatives of the thermogravimetric (DTG) curves in the Origin Pro version 8.5 program. Table 1 shows the list of materials analyzed via thermogravimetric analysis (TGA).

**Table 1:** Materials Analyzed via Thermogravimetric Analysis (TGA)

Materials	Origin	*m*_i_ (mg)^a^
Cotton	Commercially available cotton	4.6635
Microcrystalline cellulose P.A.	Labsynth Laboratory Products Ltd.	4.1133
D (+) xylose purisima (hemicellulose)	Contemporary Chemical Dynamics Ltd.	4.5043
Organosolv lignin	Lignin extracted from eucalyptus chips by the organosolv process	4.4114
Kraft lignin	Lignin extracted from sugarcane bagasse by the kraft process	4.5948
Bagasse in natura	Agricultural waste	4.5225
Bagasse without extractives	Agricultural waste	4.6734
Bagasse holocellulose	Agricultural waste	4.3153
Bagasse α-cellulose	Agricultural waste	4.5359
Bagasse lignin	Agricultural waste	4.5702
Eucalyptus in natura	Forest residue	4.3223
Eucalyptus without extractives	Forest residue	4.6958
Eucalyptus holocellulose	Forest residue	4.2087
Eucalyptus α-cellulose	Forest residue	4.2175
Eucalyptus lignin	Forest residue	4.3811

a *m*_i_: initial mass.

According to the manufacturer Labsynth, microcrystalline cellulose P.A. is a commercial product for analysis with a solid appearance and white color that is odorless, has a pH (5: 40) between 5.0 and 7.0, and is soluble in water. Xylose (C_5_H_10_O_5_) and maltose (C_12_H_22_O_11_·H_2_O) are pure sugars, which are also purchased commercially for analysis. While lignin comes from a chemical extraction process, one comes from kraft pulping from a paper and pulp company, and the other comes from organosolv pulping according to the methodology described by Rodrigues et al.^20^

## 3 Results and Discussion

Table 2 presents the results of moisture and proximate analysis, and Table 3 presents the chemical analysis for the biomass sugarcane bagasse and eucalyptus chips.

**Table 2:** Humidity and Proximate Analysis of Sugarcane and Eucalyptus Bagasse^a^

		Proximate Analysis		
Material	*U* (%)	TV (%)	TCF (%)	TC (%)
Sugarcane bagasse	9.9	79.37 ± 0.93^b^	16.31 ± 0.03	4.33 ± 0.90
Eucalyptus chips	11.2	82.18 ± 1.04	17.04 ± 1.39	0.78 ± 0.35

a *U*: moisture content; TV: volatile content; TCF: fixed carbon; TC: ash content.

b Mean ± standard deviation.

**Table 3:** Chemical Analysis of Sugarcane Bagasse and Eucalyptus

	Chemical Analysis				
Material	Extractives (%)	Lignin (%)	Holocellulose (%)	Hemicellulose (%)	α-Cellulose (%)
Sugarcane bagasse	12.75 ± 0.44^a^	11.87 ± 1.12	62.88 ± 0.83	27.64 ± 3.18	35.23 ± 3.18
Eucalyptus	11.43 ± 0.58	16.09 ± 1.48	65.75 ± 0.82	25.96 ± 2.38	39.79 ± 2.38

a Mean ± standard deviation

It is observed that the moisture content of the materials is adequate for energy use (*U* < 35%).^22,23^ Sugarcane bagasse presented higher levels of ash, extractives, and hemicellulose, while eucalyptus chips presented higher levels of volatiles, fixed carbon, lignin, holocellulose, and α-cellulose. The levels of holocellulose (65.75%) and ash (0.78%) of eucalyptus chips were close to those found by Medeiros et al.^24^ for the hybrid *Eucalyptus urophylla* × *Eucalyptus grandis*, who obtained the following results: 6.51% total extractives, 26.70% lignin, 66.12% holocellulose, and 0.67% ash. For sugarcane bagasse, the fixed carbon (16.31%), extractive (12.75%), and hemicellulose (27.64%) contents were close to those found in the literature. Fernanda Felippe Silva et al.^25^ analyzed three varieties of sugarcane bagasse and found a volatile content of around 84%, fixed carbon content of 14%, and ash content of around 2%. In the work of Rodrigues et al.,^20^ sugarcane bagasse presented 14.44% total extractive content, 20.09% total Klason lignin, 79.48% holocellulose, 28.01% hemicellulose, 51.47% α-cellulose, and 2.07% ash.

Table 4 summarizes the main parameters obtained from the thermogravimetric curves of the analyzed materials. Figure 1 shows the thermogravimetric curves, and Figure 2 shows the curves derived from thermogravimetry for each main component of the biomass: cellulose represented by cotton and microcrystalline cellulose; lignin represented by lignin resulting from the kraft extraction process and organosolv extraction; and hemicellulose represented by xylose.

**Figure 1:**
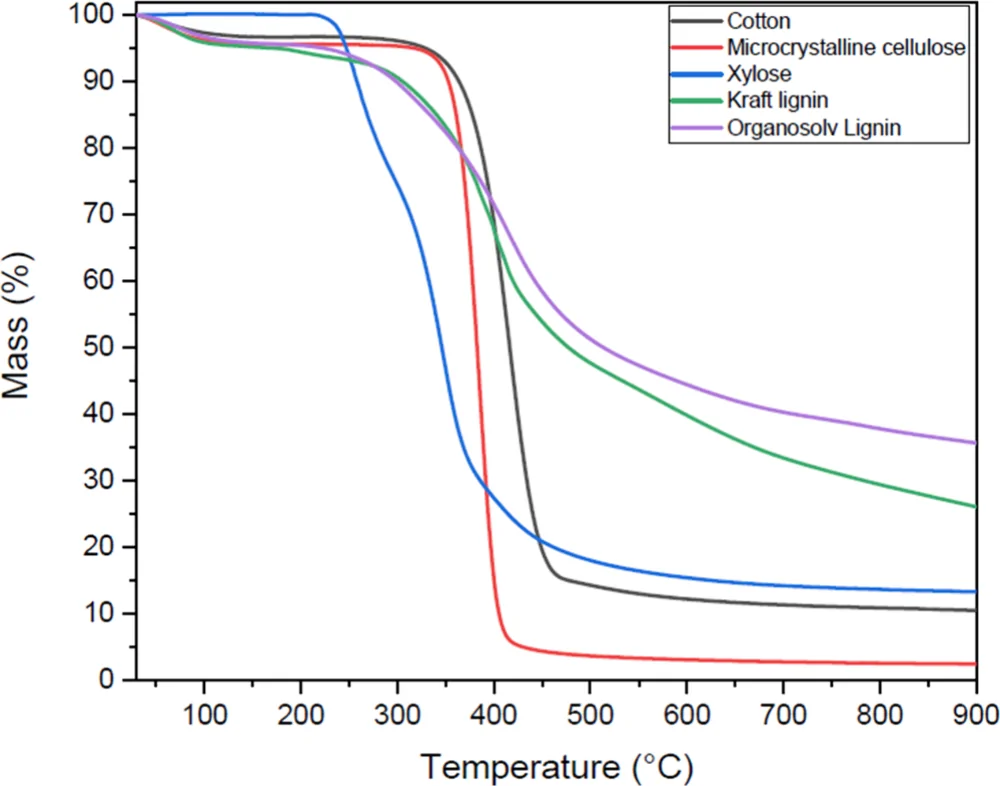
Thermogravimetric curves (TG) of biomass components.

**Figure 2:**
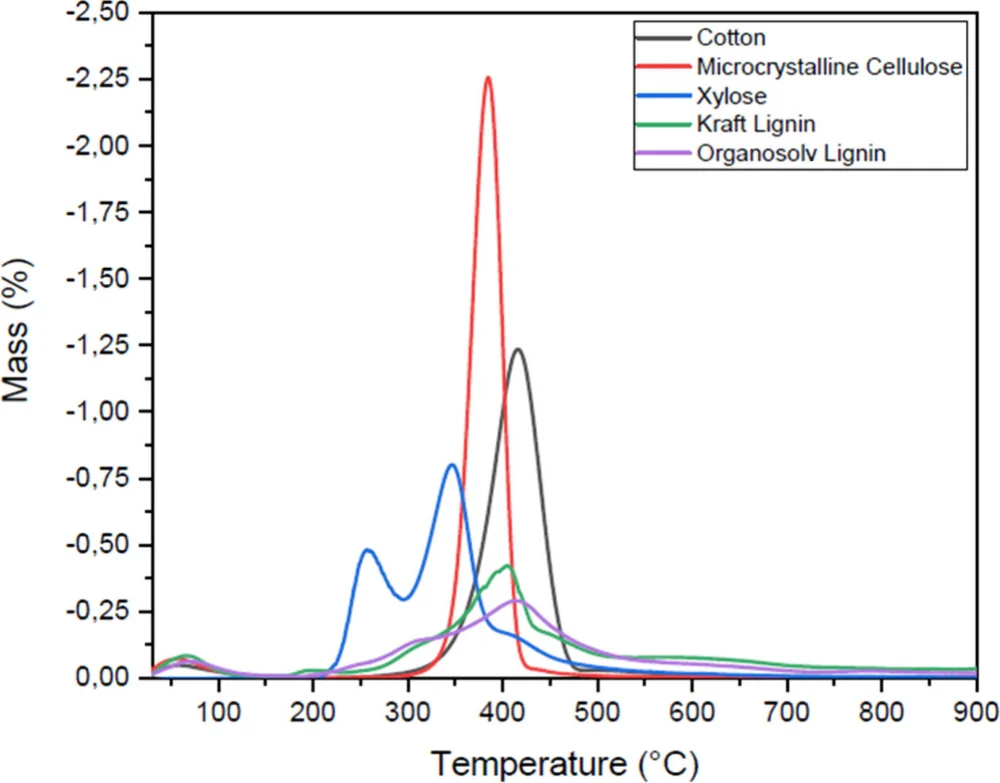
Curves derived from thermogravimetry (DTG) of biomass components.

**Table 4:** Thermogravimetric Parameters Obtained from the TG/DTG Curves of the Analyzed Materials^a^

Materials	Delta *Y* (%)	*T*_i_ (°C)	*m*_Ti_ (%)	*T*_onset_ (°C)	*m*_Tonset_ (%)	*T*_sh_ (°C)	*m*_Tsh_ (%)	*T*_max_ (°C)	*m*_Tmax_ (%)	*T*_offset_ (°C)	*m*_Toffset_ (%)
Cotton	89.50	52.89	98.59	359.24	94.28	-	-	407.93	49.58	442.76	13.08
Microcrystalline cellulose	97.50	50.81	98.15	347.09	95.07	-	-	376.20	44.64	397.75	3.71
Xylose	86.77	-	-	230.75	100.00	254.09	89.08	340.12	48.62	383.38	19.66
Organosolv lignin	64.77	62.69	98.19	278.31	93.89	306.79	87.74	405.54	67.26	468.91	50.30
Kraft lignin	75.10	58.31	97.90	328.71	90.78	194.33	94.46	399.40	64.85	558.91	39.00
Bagasse in natura	82.69	52.20	98.36	312.60	94.84	352.61	74.54	398.40	44.44	425.89	21.83
Bagasse without extractives	90.97	58.44	98.52	329.37	95.53	-	-	400.48	43.59	433.81	13.22
Bagasse holocellulose	82.33	51.51	97.98	316.69	94.14	-	-	378.28	51.04	407.22	22.83
Bagasse α-cellulose	86.85	48.73	97.37	332.25	91.95	-	-	390.07	48.96	423.86	16.09
Bagasse lignin	55.58	52.25	97.77	375.43	89.93	-	-	428.49	75.91	624.23	49.99
Eucalyptus in natura	89.41	51.51	98.28	303.54	94.76	331.10	83.87	397.70	45.02	427.14	20.06
Eucalyptus without extractives	90.11	53.59	98.43	310.62	96.11	339.43	85.44	415.05	40.42	437.45	15.22
Eucalyptus holocellulose	82.50	53.59	98.23	300.28	95.76	-	-	371.34	50.38	395.07	24.86
Eucalyptus α-cellulose	80.56	50.81	98.36	325.21	94.43	-	-	376.89	52.29	406.40	23.79
Eucalyptus lignin	52.34	53.65	98.25	354.97	94.95	-	-	420.84	79.25	540.30	56.81

a Parameters of the thermogravimetric curves of the analyzed materials: delta *Y*, percentage of total mass loss in relation to the initial mass; *T*_i_, initial decomposition temperature; *m*_Ti_, percentage of mass at the beginning of decomposition; *T*_onset_, onset temperature; *m*_Tonset_, mass remaining at the onset temperature; *T*_sh_, deflection temperature before the maximum decomposition point; *m*_Tsh_, mass remaining at the deflection temperature; *T*_max_, maximum decomposition temperature; *m*_Tmax_, mass remaining at the maximum decomposition temperature; *T*_offset_, temperature at which stabilization of the biomass decomposition occurs; and *m*_Toffset_, final mass at which stabilization of the thermal decomposition of the biomass occurs.

The thermogravimetric behavior of the analyzed materials revealed clear differences in thermal stability among the main lignocellulosic constituents, providing the basis for interpreting the thermal decomposition of eucalyptus wood chips and sugarcane bagasse (Figures 1 and 2; Table 4). Moisture loss began at approximately 50 °C for all materials, as indicated by the initial DTG peak below 100 °C. However, the most significant differences were observed during the devolatilization stage, in which the degradation profile was strongly influenced by the chemical structure of each component.

Lignin samples (bagasse lignin, chip lignin, kraft lignin, and organosolv lignin) exhibited lower delta *Y* values, broader degradation intervals, and higher residual masses than the carbohydrate-derived materials (Table 4). These characteristics indicate that lignin is the most thermally resistant constituent of lignocellulosic biomass since its heterogeneous aromatic structure promotes gradual bond cleavage over a wide temperature range rather than rapid decomposition. In contrast, cellulose and xylose decomposed within narrower temperature intervals, resulting in sharper TG and DTG profiles (Figures 1 and 2).

The thermogravimetric parameters also showed that only in natura sugarcane bagasse, in natura eucalyptus, extractive-free eucalyptus, xylose, kraft lignin, and organosolv lignin exhibited deflection temperatures prior to the maximum degradation temperature (Table 4). Moreover, when comparing equivalent biomass fractions, sugarcane bagasse generally exhibited higher characteristic temperatures than eucalyptus, indicating greater thermal stability.

The degradation stages observed in this study are consistent with the pyrolysis behavior commonly reported for lignocellulosic biomass under an inert atmosphere. In general, thermal decomposition occurs in three stages: (i) moisture evaporation below approximately 250 °C; (ii) decomposition of hemicellulose and cellulose between 250 and 400 °C; and (iii) gradual lignin degradation above 400 °C, leading to char formation and ash production.^13,28,29^ Postawa et al.^30^ proposed a more detailed classification consisting of dehydration (up to 120 °C), active pyrolysis (120–340 °C), passive pyrolysis (340–480 °C), and residual pyrolysis, which is also consistent with the degradation profiles observed in the present study. Extractives are mainly degraded between 200 and 400 °C; however, their decomposition cannot be clearly distinguished from that of the structural lignocellulosic fractions because of the overlap between degradation events.^8,21^ In addition, the identification of intermediate compounds in thermogravimetric curves depends on both the heating rate and the nature of the analyzed sample.^26,27^

The reference compounds further clarify the contribution of each structural component to the thermal decomposition of lignocellulosic biomass. As shown in Figures 1 and 2, cotton and microcrystalline cellulose exhibited abrupt mass-loss events accompanied by narrow DTG peaks, whereas kraft and organosolv lignins degraded over a much broader temperature range (approximately 270–560 °C). Xylose showed an intermediate behavior, with degradation beginning at lower temperatures than that of cellulose. These contrasting profiles demonstrate that the sharp degradation peaks commonly observed in whole biomass are predominantly associated with carbohydrate decomposition, whereas the broad high-temperature degradation tail mainly reflects the progressive decomposition of lignin.

To establish the origin of these degradation events, we discuss the thermal behavior of the isolated reference compounds in the following sections. Microcrystalline cellulose and cotton were used as cellulose models (Figures 3 and 4). Kraft and organosolv lignins represented the lignin fraction (Figures 5 and 6), and xylose was used as a model compound for hemicellulose (Figure 7). Understanding the thermal behavior of these individual components provides the mechanistic framework for interpreting the TG and DTG profiles of eucalyptus wood chips and sugarcane bagasse.

**Figure 3:**
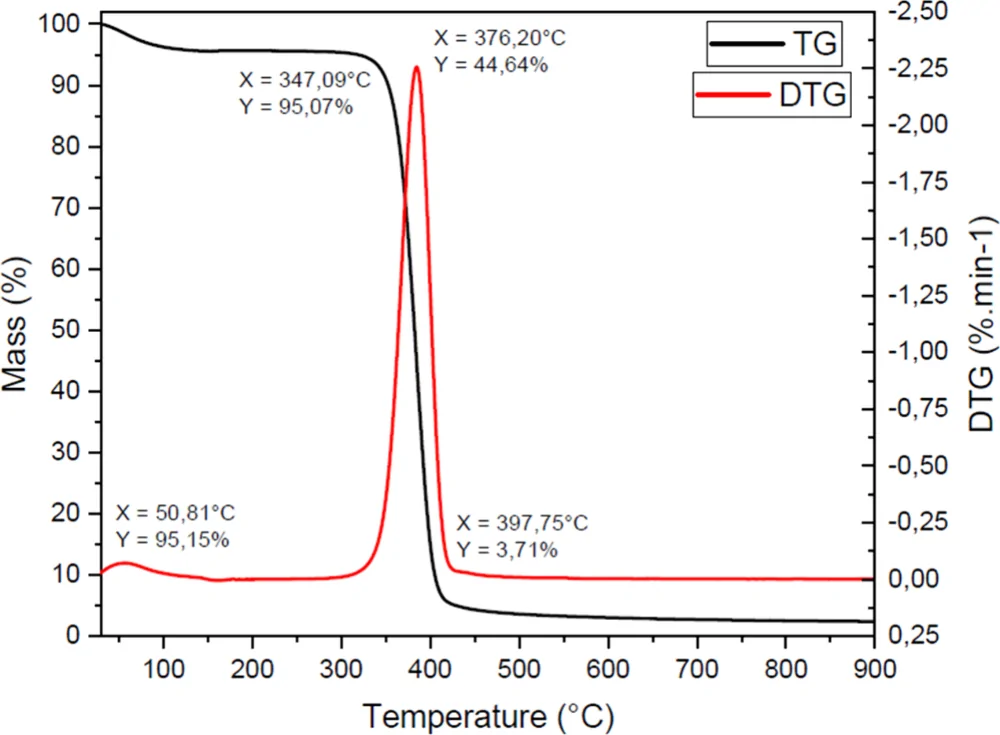
Thermogravimetric (TG) and derivative thermogravimetric (DTG) curves of microcrystalline cellulose.

**Figure 4:**
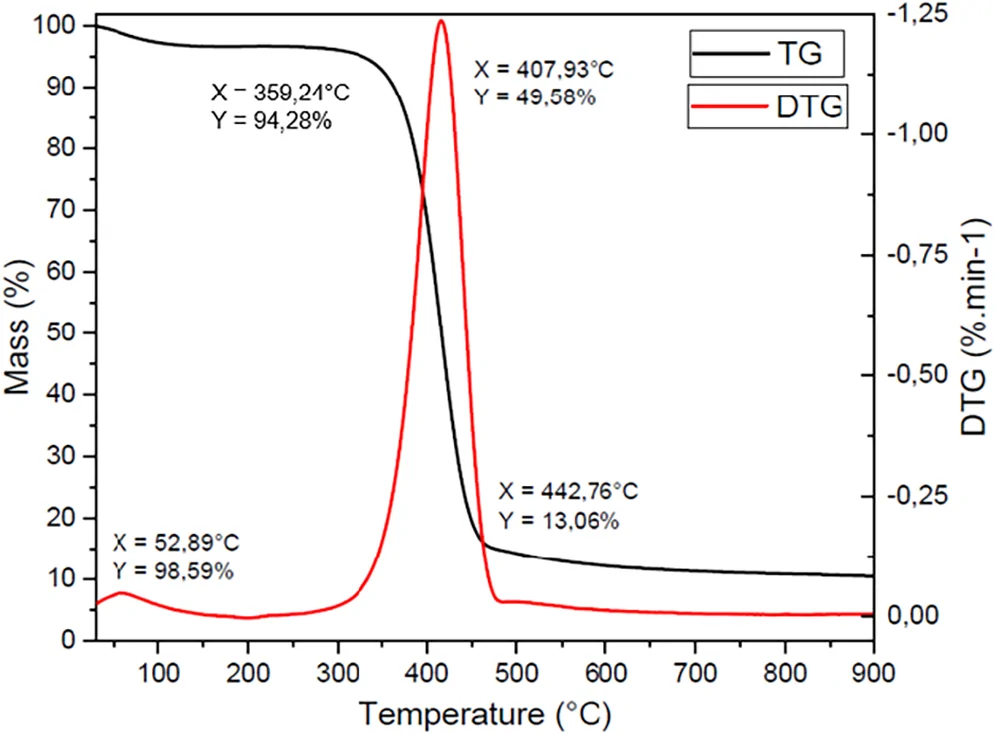
Thermogravimetric (TG) and derivative thermogravimetric (DTG) curves of cotton.

**Figure 5:**
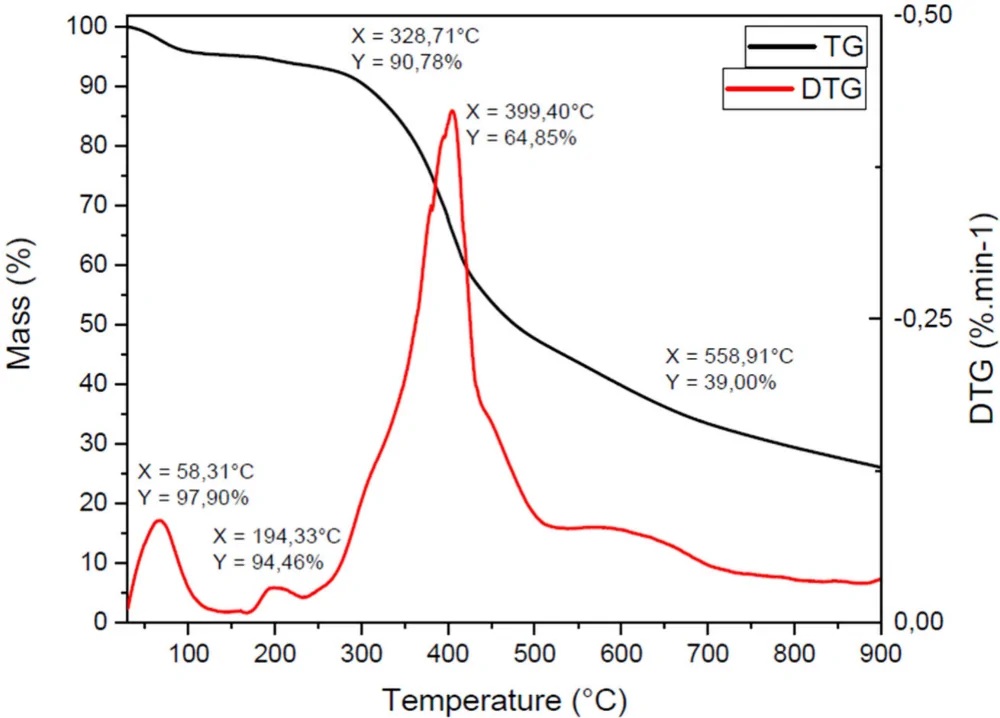
Thermogravimetric (TG) and derivative thermogravimetric (DTG) curves of kraft lignin.

**Figure 6:**
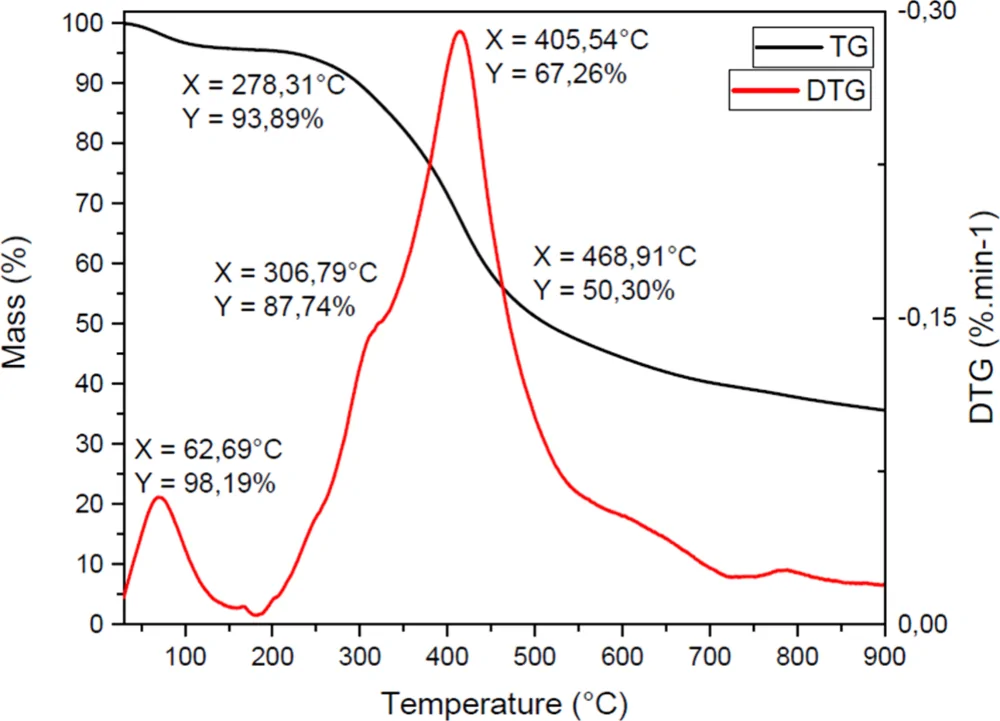
Thermogravimetric (TG) and derivative thermogravimetric (DTG) curves of organosolv lignin.

**Figure 7:**
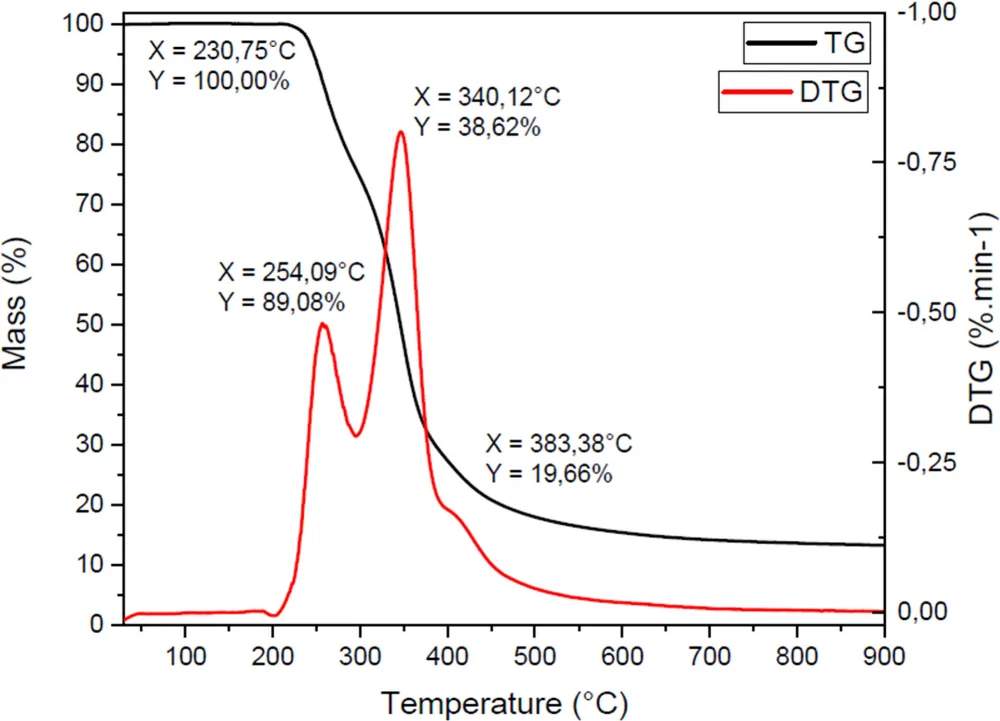
Thermogravimetric (TG) and derivative thermogravimetric (DTG) curves of xylose.

In order to elucidate the thermal degradation behavior of each of the biomass components analyzed, the thermogravimetric curves are discussed individually. The thermogravimetric analysis (TG and DTG curves) is presented as a function of temperature for microcrystalline cellulose (Figure 3) and cotton (Figure 4).

The cellulose reference materials exhibited the typical thermal degradation pattern of crystalline cellulose, characterized by a single, sharp degradation event concentrated within a narrow temperature interval (Figures 3 and 4). The relatively regular arrangement of β-1,4-glucose chains and the hydrogen-bonded crystalline domains of cellulose contribute to this concentrated thermal response.^31,32^ This behavior confirms that cellulose is responsible for the rapid mass-loss stage commonly observed during the pyrolysis of lignocellulosic biomass.

Microcrystalline cellulose and cotton started to degrade at 347.09 and 359.24 °C (*T*_onset_), respectively, reaching their maximum degradation rates at 376.20 and 407.93 °C (*T*_max_), followed by complete stabilization at 397.75 and 442.76 °C (*T*_offset_) (Table 4). The narrow degradation interval agrees with the behavior reported by Díez et al.^21^ and reflects the relatively homogeneous molecular structure of cellulose.

The degradation temperatures obtained in this study are also consistent with previous investigations. Anca-Couce et al.^33^ reported maximum decomposition rates at 328.3 ± 9.2 °C, while Chen et al.^2^ demonstrated that cellulose remains relatively stable below 300 °C and becomes highly reactive above this temperature due to thermal depolymerization. Likewise, Osman et al.^34^ observed that nearly 80% of cellulose conversion occurs between 350 and 400 °C, confirming the high degradation rate within this temperature interval. These results demonstrate that cellulose is the main contributor to the pronounced DTG peak typically observed between 300 and 400 °C during biomass pyrolysis.

Figure 5 shows the thermogravimetric analysis of kraft lignin, and Figure 6 shows the analysis of organosolv lignin.

Unlike cellulose, lignin exhibited a much broader degradation profile, indicating that its thermal decomposition occurs through multiple overlapping reactions rather than a single degradation event (Figures 5 and 6). This broad profile is consistent with the heterogeneous aromatic structure of lignin, in which guaiacyl, syringyl, and *p*-hydroxyphenyl units are connected by different ether and carbon–carbon linkages with varying thermal stability.^31,32^ The undulations observed in the DTG curves of both kraft and organosolv lignins suggest the occurrence of several decomposition stages associated with the progressive cleavage of different oxygen-containing functional groups.

Kraft and organosolv lignins initiated thermal degradation at 328.71 and 278.31 °C (*T*_onset_), exhibited deflection temperatures (*T*_sh_) at 194.33 and 306.76 °C, reached maximum degradation at 399.40 and 405.54 °C (*T*_max_), and completed degradation at 558.91 and 468.91 °C (*T*_offset_), respectively (Table 4).

The broad degradation interval agrees with previous studies reporting lignin decomposition over a wide temperature range because of its highly heterogeneous aromatic structure.^8,36,37^ Kristanto et al.^35^ also observed multiple overlapping DTG peaks at approximately 270, 310, and 380 °C, corresponding to successive lignin pyrolysis reactions.

The persistence of lignin-derived material at higher temperatures is associated with its heterogeneous aromatic structure and the formation of increasingly carbon-rich structures through secondary condensation, contributing to the higher residual mass observed for lignin.^31,38^ Consequently, lignin contributes predominantly to the high-temperature degradation tail during biomass pyrolysis. Among the isolated lignins, organosolv lignin exhibited a slightly higher maximum degradation temperature and residual mass than kraft lignin, indicating greater thermal stability. This difference is mainly attributed to the extraction process. Because organosolv pulping causes fewer structural modifications, its lignin remains closer to native lignin, whereas kraft lignin undergoes substantial chemical alterations, including changes in the syringyl/guaiacyl ratio and the formation of additional phenolic structures.^20,39–42^ These structural differences explain the distinct TG and DTG profiles observed for both lignins.

Figure 7 shows the TG and DTG curves of xylose as a function of temperature, representing hemicelluloses.

Xylose exhibited an intermediate thermal behavior between cellulose and lignin (Figure 7), decomposing at a lower temperature than cellulose while presenting a broader degradation profile. Thermal degradation started at 230.75 °C (*T*_onset_), followed by a first DTG peak at 254.09 °C (*T*_sh_) and a second, more intense peak at 340.12 °C (*T*_max_). Mass stabilization occurred at 383.38 °C (*T*_offset_) (Table 4).

The presence of two degradation peaks reflects the complex degradation pathway of xylose and agrees with previous thermogravimetric studies. Felsner^43^ also observed two DTG peaks at approximately 215 and 314 °C, whereas Zanatta et al.^18^ reported hemicellulose degradation between 208 and 447 °C using xylose as a model compound. Although the maximum degradation temperature observed here was slightly higher, both the degradation interval and the residual mass were similar.

The lower thermal stability of xylose is associated with the amorphous and highly branched nature of hemicelluloses.^2^ The presence of diverse linkages and substituents contributes to a less uniform structure than that of cellulose, favoring earlier fragmentation and overlapping degradation events.^32,44^ Furthermore, the thermal stability of monosaccharides depends on their cyclic molecular conformation, with xylose exhibiting intermediate stability among the main monosaccharides.^43^

Overall, the isolated reference compounds clearly demonstrate that the sharp degradation peak observed in lignocellulosic biomass originates predominantly from carbohydrate decomposition, whereas lignin governs the slower degradation observed at higher temperatures.

Figures 8–13 present the TG and DTG curves of eucalyptus and sugarcane bagasse, respectively, including the in natura biomass and the sequentially isolated fractions (extractive-free biomass, holocellulose, α-cellulose, and lignin).

**Figure 8:**
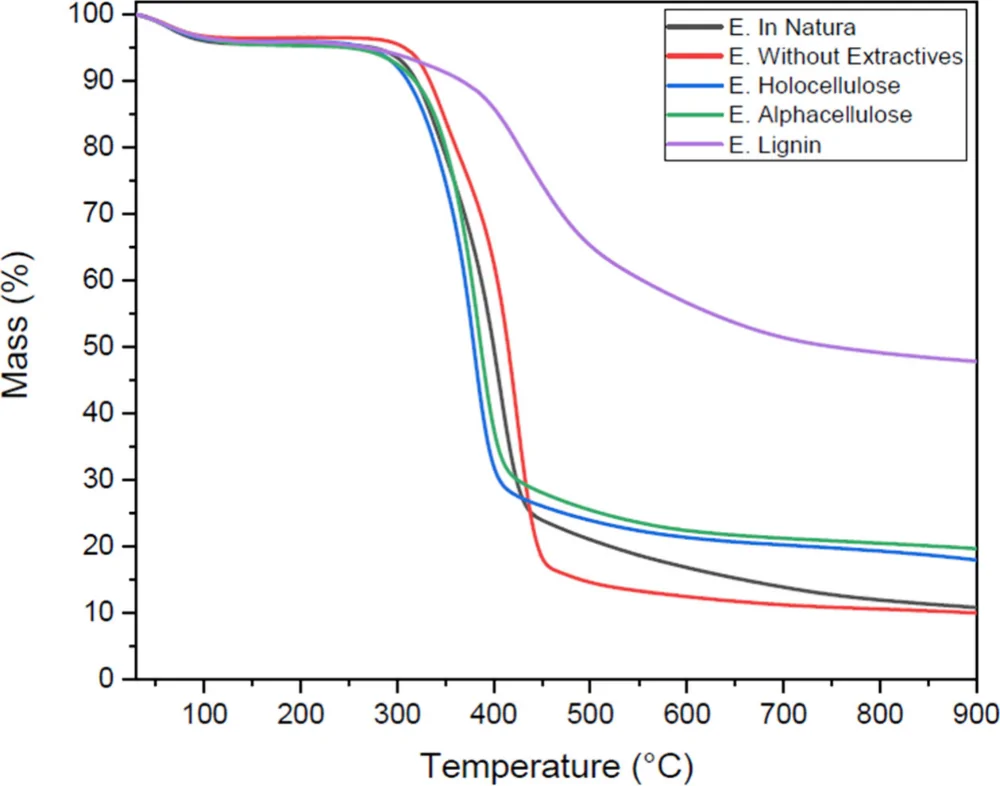
Thermogravimetric curves (TG) of eucalyptus as a function of temperature.

Comparison of these profiles demonstrates that the thermal decomposition behavior of both biomasses is governed by the progressive contribution of their lignocellulosic constituents, with the thermal profile of the in natura biomass resulting from the overlap of the degradation events of extractives, hemicellulose, cellulose, and lignin.

For both biomasses, the TG and DTG profiles of the extractive-free samples remained similar to those of the corresponding in natura materials, indicating that extractives have a limited influence on the overall degradation pathway but affect the initial stages of devolatilization. In eucalyptus, the DTG curve of the extractive-free biomass was slightly shifted toward higher temperatures than that of the in natura sample, indicating a modest increase in thermal stability after extractive removal. Similarly, in sugarcane bagasse, the in natura, extractive-free, and holocellulose fractions exhibited comparable DTG profiles; however, the shoulder corresponding to the deflection temperature (*T*_sh_), clearly observed in the in natura biomass, gradually decreased after extractive removal and became only a slight curvature in the holocellulose fraction. This behavior suggests that low-molecular-weight compounds and part of the hemicellulose contribute to the initial degradation events, broadening the DTG profile. Since extractives decompose over a wide temperature interval (approximately 200–400 °C), their degradation partially overlaps with that of hemicellulose and cellulose, contributing to the complexity of the thermal profile.^8,21^

This interpretation was quantitatively supported by Gaussian deconvolution of the DTG curves, as shown in Table 5. The fitted models showed excellent agreement with the experimental data, with adjusted *R* ^2^ values ranging from 0.939 to 0.996, confirming that the main degradation region of the in natura biomasses is composed of overlapping thermal events rather than a single decomposition process. For in natura sugarcane bagasse, two overlapping peaks centered at 365 and 411 °C accounted for 63.3 and 36.7% of the total peak area, respectively. Following extractive removal, only a single degradation peak centered at 398 °C remained, representing 100% of the degradation area. Likewise, in natura eucalyptus exhibited two peaks with nearly equal contributions (50.2 and 49.8%), whereas after extractive removal the contribution of the first event decreased to 41.9% and the second increased to 58.1%. In the holocellulose fraction, only one degradation peak centered at 372 °C was identified. The progressive reduction and eventual disappearance of the first deconvoluted event quantitatively confirms that the DTG shoulder originates from overlapping degradation processes associated mainly with extractives and hemicellulose, whereas the remaining dominant peak corresponds predominantly to cellulose degradation.

**Table 5:** Gaussian Deconvolution Parameters Obtained from DTG Curves of Eucalyptus Wood and Sugarcane Bagasse and Their Structural Fractions

Material	Adjusted *R* ^2^	Peak 1 *T* (°C)	Peak 1 Area	Peak 1 (%)^a^	Peak 2 *T* (°C)	Peak 2 Area	Peak 2 (%)^a^
Bagasse in natura	0.99573	365	43.68	63.32	411	25.3	36.68
Bagasse without extractives	0.96659	-	-	-	398	79.9	100
Eucalyptus in natura	0.99058	355	34.01	50.2	407	33.69	49.77
Eucalyptus without extractives	0.99346	363	33.09	41.86	423	45.95	58.14
Extractive-free eucalyptus wood	0.93896	-	-	-	372	60.37	100

a Relative contribution of each deconvoluted peak, calculated as the percentage of the total peak area.

The Gaussian model was selected as an empirical and stable approach for resolving partially overlapping DTG maxima and enabling a consistent comparison among the samples. Deconvolution of overlapping DTG signals can help distinguish thermal contributions that are not clearly resolved in the experimental profile, providing estimates of their characteristic temperatures and relative areas.^45,46^ The method assumes that the experimental DTG profile can be represented by the additive contribution of overlapping components with each component approximated by a Gaussian function. However, this is a mathematical approximation rather than a mechanistic assumption, and the fitted peaks should not be interpreted as direct evidence of individual chemical reactions.^45^ The main limitations are the model-dependent nature of the deconvolution and the sensitivity of the fitted parameters to the baseline, fitting range, number of components, and assumed peak shape; particularly, symmetric Gaussian functions may introduce deviations when the experimental peaks are asymmetric.^46^ Therefore, the peak temperatures reported in Table 5 should be regarded as characteristic temperatures of the fitted contributions under the experimental conditions employed. Despite these limitations, the high adjusted *R* ^2^ values and the consistent trends observed among the samples support the use of Gaussian deconvolution as a useful comparative tool for resolving overlapping features in DTG curves.

The transition from extractive-free biomass to holocellulose produced more pronounced changes in both of the biomasses. In eucalyptus, the DTG profile became considerably narrower and the intermediate peak preceding the maximum degradation peak disappeared, whereas in sugarcane bagasse, this feature progressively diminished until only a slight left-side inflection remained. These observations indicate that the thermal heterogeneity of the native biomass results from the overlapping degradation of the extractives, hemicellulose, cellulose, and lignin. The progressive simplification of the DTG profile after sequential extraction demonstrates that each structural constituent contributes to the overall degradation profile. In particular, extractives and hemicellulose are primarily responsible for the shoulder preceding the maximum degradation rate, whereas cellulose dominates the principal devolatilization events. Lignin, because of its highly heterogeneous aromatic structure, extends degradation over a broad temperature interval, contributing mainly to the high-temperature tail of the DTG profile. Consequently, after lignin removal, the devolatilization process becomes increasingly dominated by polysaccharides, resulting in a more defined DTG event.

The α-cellulose fractions of eucalyptus and sugarcane bagasse exhibited very similar DTG profiles, characterized by a single and sharp degradation peak. In sugarcane bagasse, the *T*_sh_ event was no longer observed, leaving only the maximum degradation peak, a behavior comparable to that previously observed for cotton and microcrystalline cellulose. This result reflects the relatively homogeneous and partially crystalline structure of cellulose, which decomposes within a narrow temperature interval. In contrast, lignin exhibited the broadest degradation profile in both biomasses, with thermal decomposition extending over a wide temperature range and maximum degradation occurring at higher temperatures, consistent with its heterogeneous aromatic structure and with previous reports in the literature.^8,21^

The thermal profile of the in natura biomasses can therefore be interpreted as the superposition of the degradation events associated with their individual constituents rather than as a single thermal process. For eucalyptus (Figures 8, 9, and 10), the initial mass loss occurred at 51.51 °C (*T*_i_), corresponding to moisture evaporation. Thermal degradation began at 303.54 °C (*T*_onset_), marking the onset of decomposition of the structural carbohydrates. The Gaussian deconvolution resolved the main degradation region into two overlapping events centered at approximately 355 and 407 °C, with relative contributions of 50.2 and 49.8%, respectively. These results indicate that the lower-temperature event is associated with the overlapping degradation of extractives and hemicellulose and the onset of cellulose decomposition, whereas the second event is predominantly attributed to cellulose degradation. The DTG shoulder observed at 331.10 °C is therefore interpreted as the manifestation of these overlapping degradation processes rather than as an independent decomposition event. At higher temperatures, lignin contributed to the gradual decline of the DTG profile, while mass loss stabilized at 427.14 °C (*T*_offset_), corresponding to the final carbonization stage, leaving char and ash as the remaining solid products. Sugarcane bagasse (Figures 11, 12, and 13) exhibited a similar decomposition sequence. The initial mass-loss event occurred at 52.20 °C due to moisture evaporation, followed by the onset of degradation at 312.60 °C (*T*_onset_). Gaussian deconvolution separated the principal degradation region into two overlapping events centered at approximately 365 and 411 °C, contributing to 63.3 and 36.7% of the total degradation area, respectively. After extractive removal, only one degradation peak remained, confirming that the lower-temperature event was associated with the overlapping contribution of extractives and hemicellulose. The shoulder observed at 352.61 °C (*T*_sh_) was predominantly associated with hemicellulose decomposition, whereas the maximum degradation rate occurred at 398.40 °C (*T*_max_), corresponding mainly to cellulose degradation with the progressive contribution of lignin. Mass loss stabilized at 425.89 °C (*T*_offset_). Compared with eucalyptus, sugarcane bagasse showed slightly higher *T*_onset_ and *T*_max_ values, suggesting marginally greater thermal stability of its carbohydrate fraction, which may be associated with differences in the chemical composition and structural organization of the lignocellulosic matrix. Similar thermal behavior has been reported for sugarcane bagasse by Zanatta et al., who observed degradation between 270 and 480 °C with *T*_max_ at approximately 384 °C.^18^

**Figure 9:**
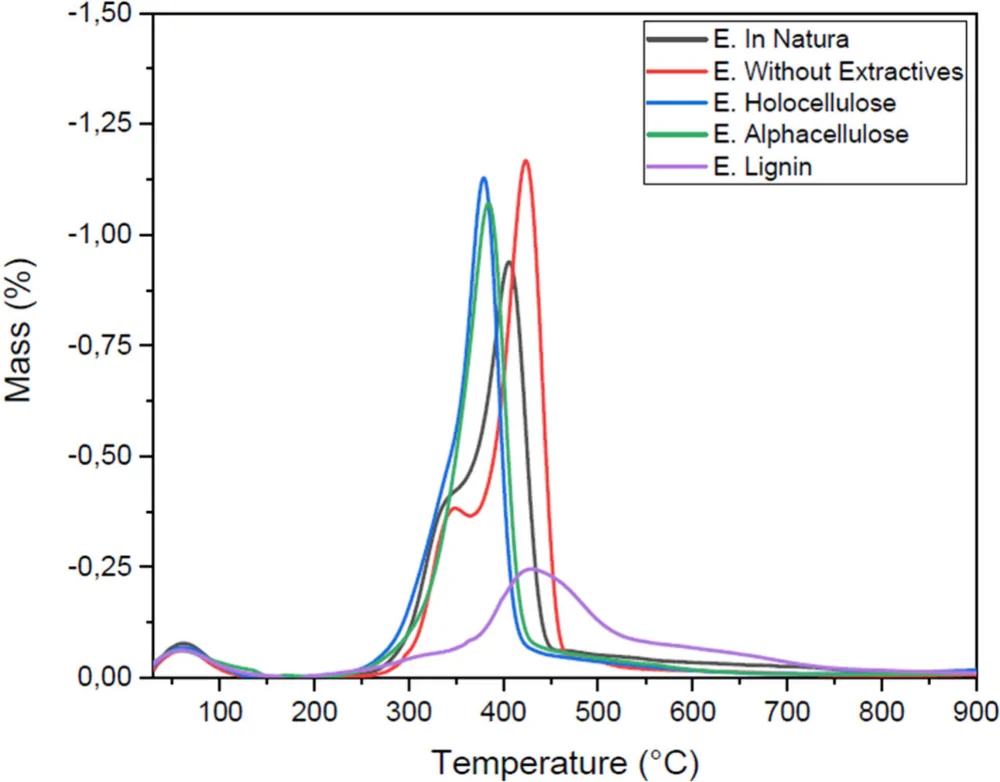
Curves derived from thermogravimetry (DTG) of eucalyptus as a function of temperature.

**Figure 10:**
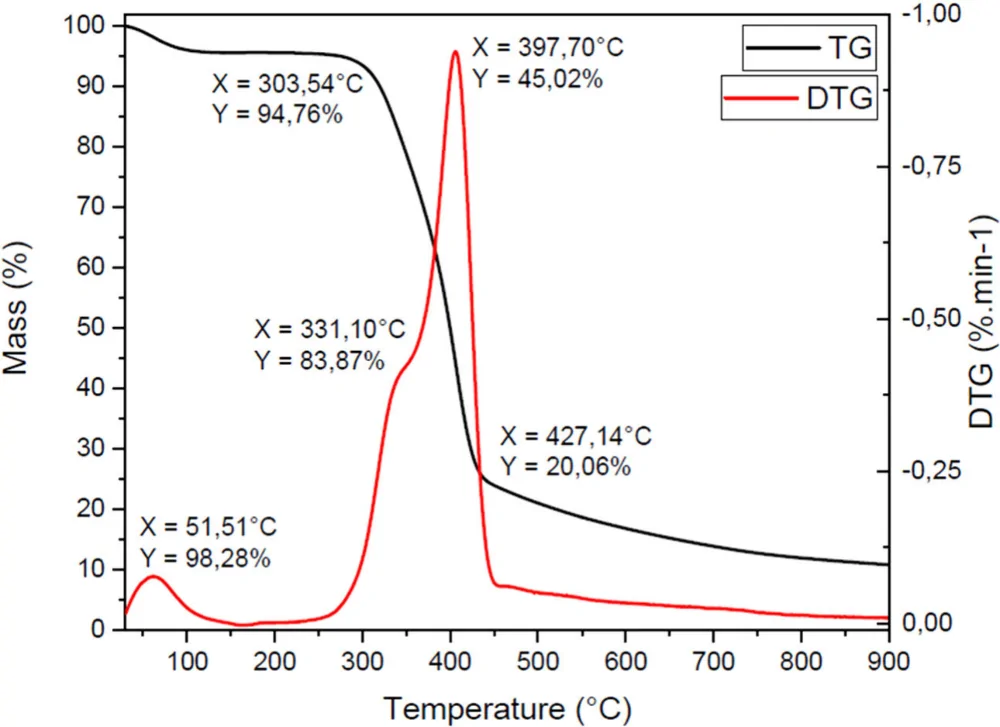
Thermogravimetric (TG) and derivative thermogravimetric (DTG) curves of in natura eucalyptus as a function of temperature.

**Figure 11:**
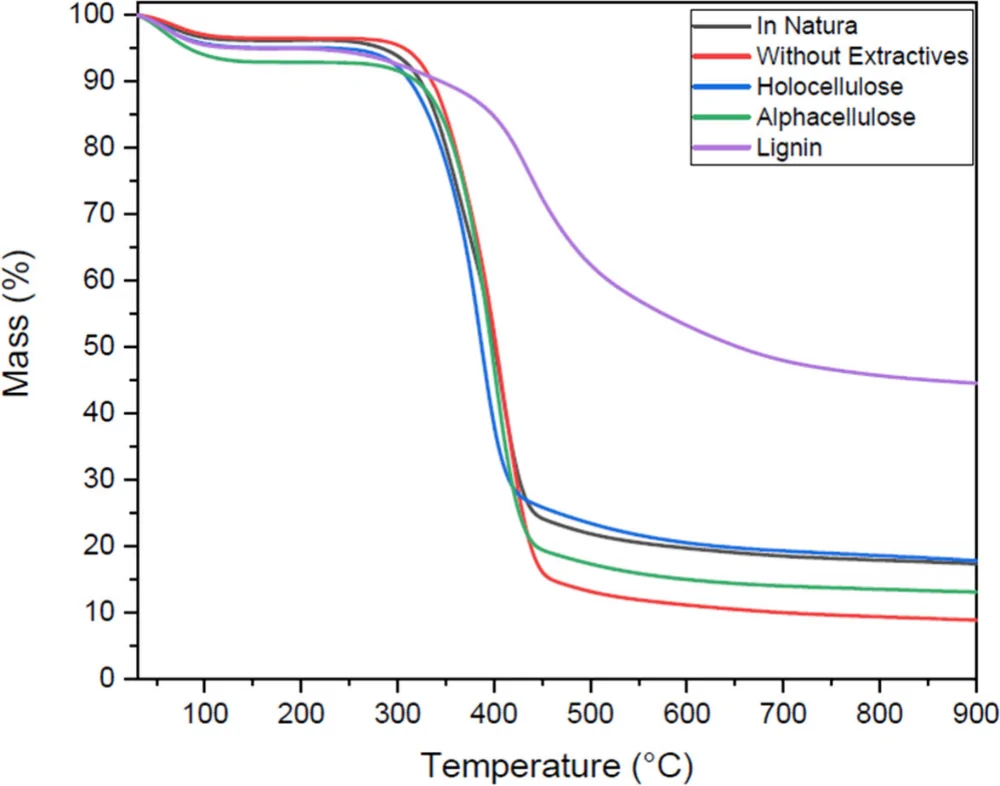
Thermogravimetric curves (TG) of sugarcane bagasse as a function of temperature.

**Figure 12:**
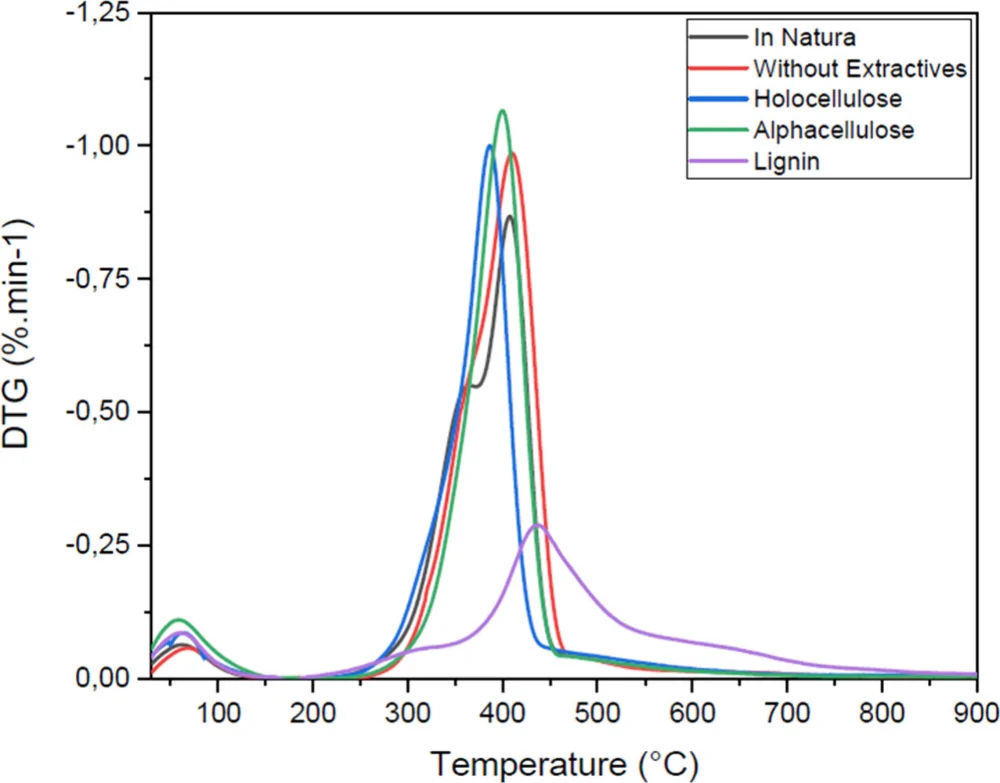
Curves derived from thermogravimetry (DTG) of sugarcane bagasse as a function of temperature.

**Figure 13:**
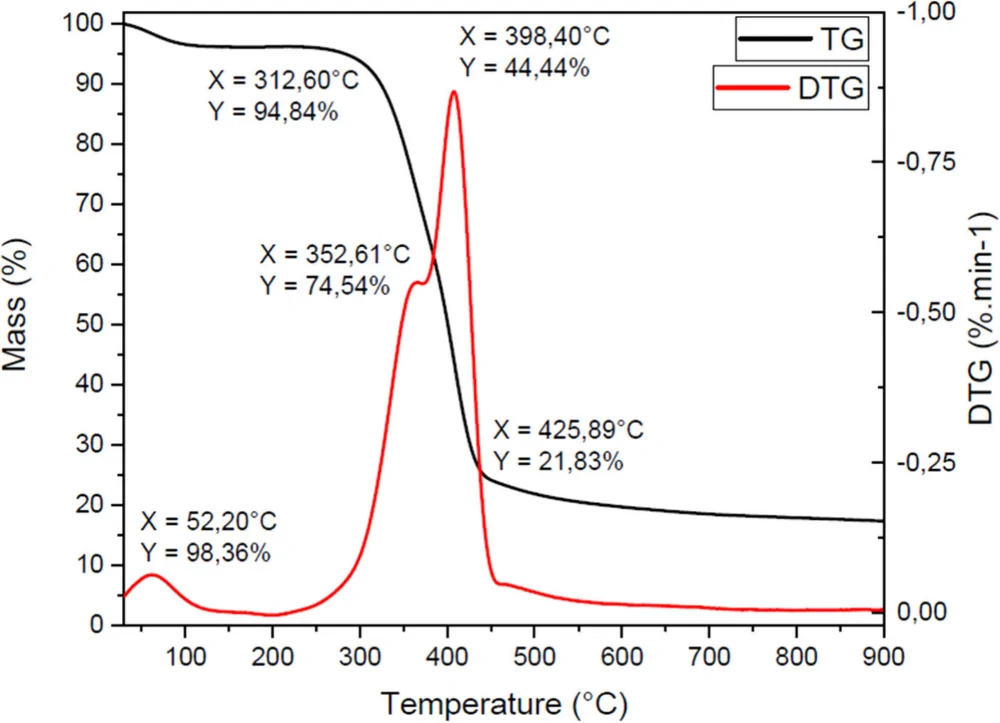
Thermogravimetric (TG) and derivative thermogravimetric (DTG) curves of in natura sugarcane bagasse.

Figures 14 and 15 present the TG and DTG curves of extractive-free eucalyptus and sugarcane bagasse, respectively.

**Figure 14:**
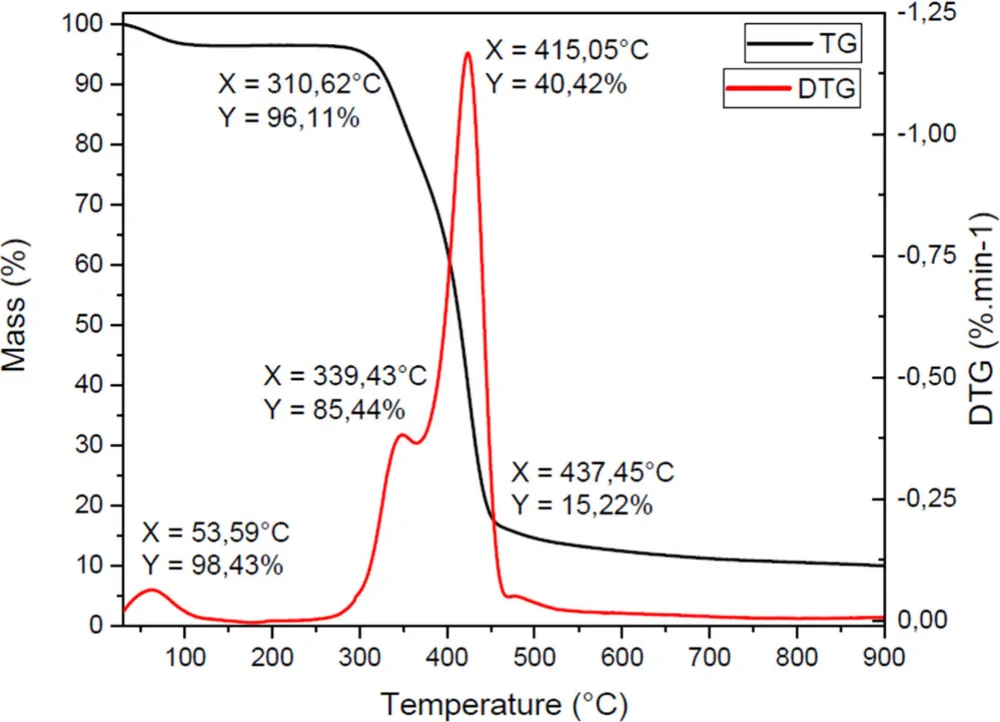
Thermogravimetric (TG) and derivative thermogravimetric (DTG) curves of eucalyptus without extractives as a function of temperature.

**Figure 15:**
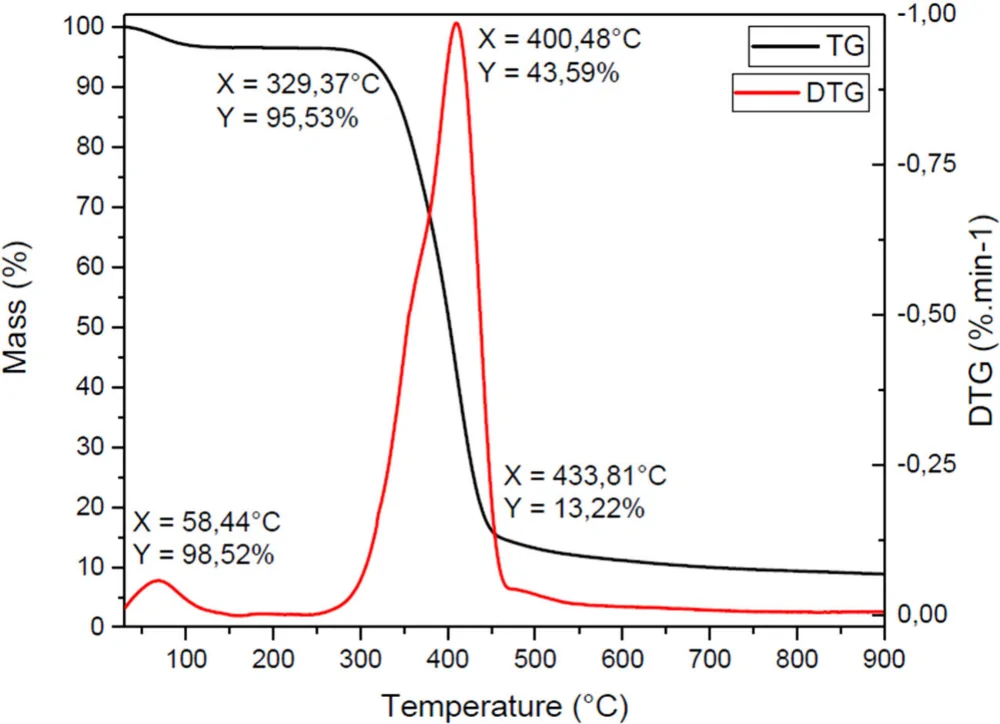
Thermogravimetric (TG) and derivative thermogravimetric (DTG) curves of sugarcane bagasse without extractives.

Comparison with the corresponding in natura biomasses demonstrates that extractive removal modified the initial stages of thermal decomposition in both materials, resulting in slightly higher degradation temperatures and, consequently, greater thermal stability.

For eucalyptus (Figure 14), the TG and DTG profiles of the extractive-free biomass remained similar to those of the in natura material, indicating that extractives have a limited influence on the overall decomposition pathway. However, all characteristic thermal events were shifted toward higher temperatures after extractive removal. The onset of degradation (*T*_onset_) increased from 303.54 to 310.62 °C, the first DTG peak shifted from 331.10 to 339.43 °C, the maximum degradation temperature (*T*_max_) increased from 397.70 to 415.05 °C, and the final degradation stage (*T*_offset_) shifted from 427.14 to 437.45 °C. In addition, the maximum degradation temperature increased by 2.48%, indicating a modest improvement in thermal stability after the removal of extractives. These results suggest that low-molecular-weight compounds present in the native biomass promote earlier degradation events, whereas their removal delays the decomposition of the remaining lignocellulosic matrix. Similar increases in the initial degradation temperature after extractive removal have been reported for eucalyptus and pine species, confirming that extractives contribute to the early stages of thermal decomposition.^47–49^

A similar trend was observed for sugarcane bagasse (Figure 15). Although the overall TG and DTG profiles remained comparable to those of the in natura biomass, the DTG curve of the extractive-free sample no longer exhibited the shoulder corresponding to the deflection temperature (*T*_sh_) preceding the maximum degradation peak. The disappearance of this thermal event suggests that part of the compounds responsible for the initial mass-loss process was removed during extraction. Since extractives begin to decompose at approximately 200 °C, their thermal degradation partially overlaps with that of hemicellulose, contributing to the shoulder observed in the in natura DTG profile.^8,21^ Consequently, extractive removal simplifies the thermal profile and makes the degradation of the structural carbohydrates more evident.

A similar behavior observed for both biomasses demonstrates that, although extractives represent a relatively small fraction of the lignocellulosic matrix, they significantly influence the initial stages of thermal decomposition by contributing to overlapping degradation events. Their removal increases the apparent thermal stability of the remaining material and improves the definition of the DTG profile, reinforcing that the thermal behavior of lignocellulosic biomass depends not only on the major structural polymers but also on the presence of low-molecular-weight compounds that participate in the early stages of devolatilization.

The eucalyptus holocellulose and α-cellulose profiles are presented in Figures 16 and 17, respectively, while the corresponding lignin profile is shown in Figure 18. For sugarcane bagasse, the holocellulose, α-cellulose, and lignin profiles are presented in Figures 19, 20, and 21, respectively.

**Figure 16:**
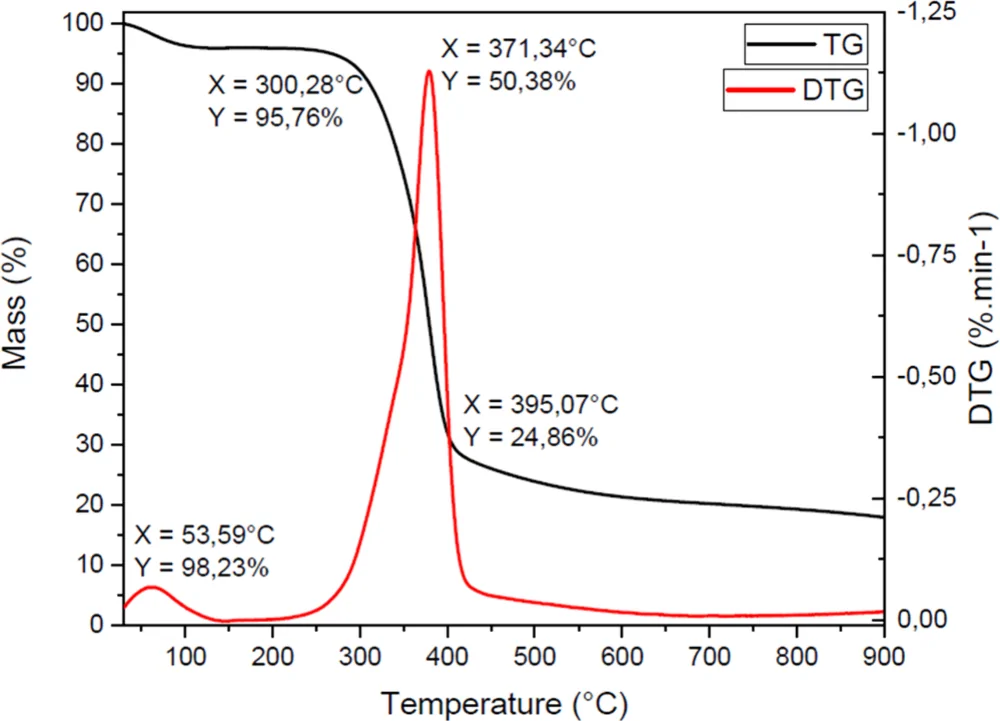
Thermogravimetric (TG) and derivative thermogravimetric (DTG) curves of eucalyptus holocellulose as a function of temperature.

**Figure 17:**
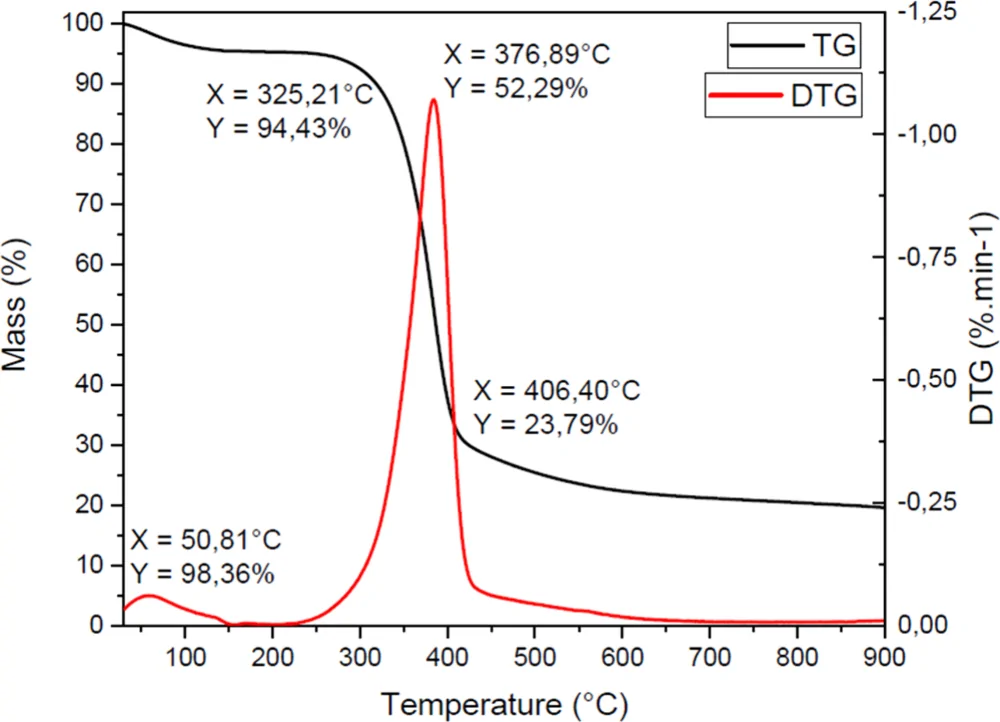
Thermogravimetric (TG) and derivative thermogravimetric (DTG) curves of eucalyptus α-cellulose as a function of temperature.

**Figure 18:**
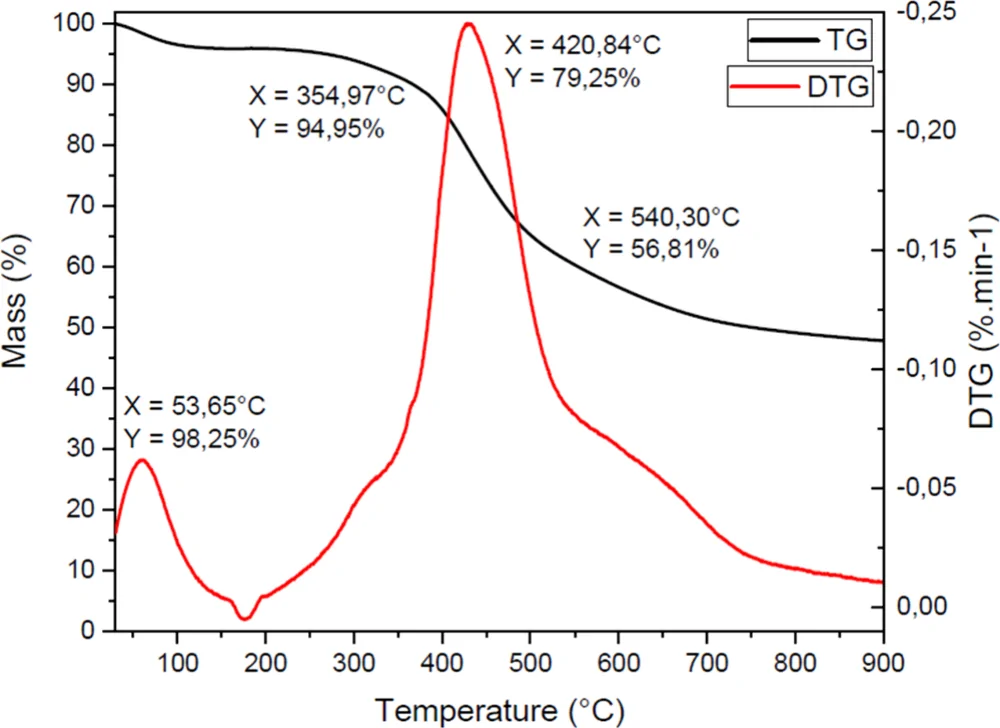
Thermogravimetric (TG) and derivative thermogravimetric (DTG) curves of eucalyptus lignin as a function of temperature.

**Figure 19:**
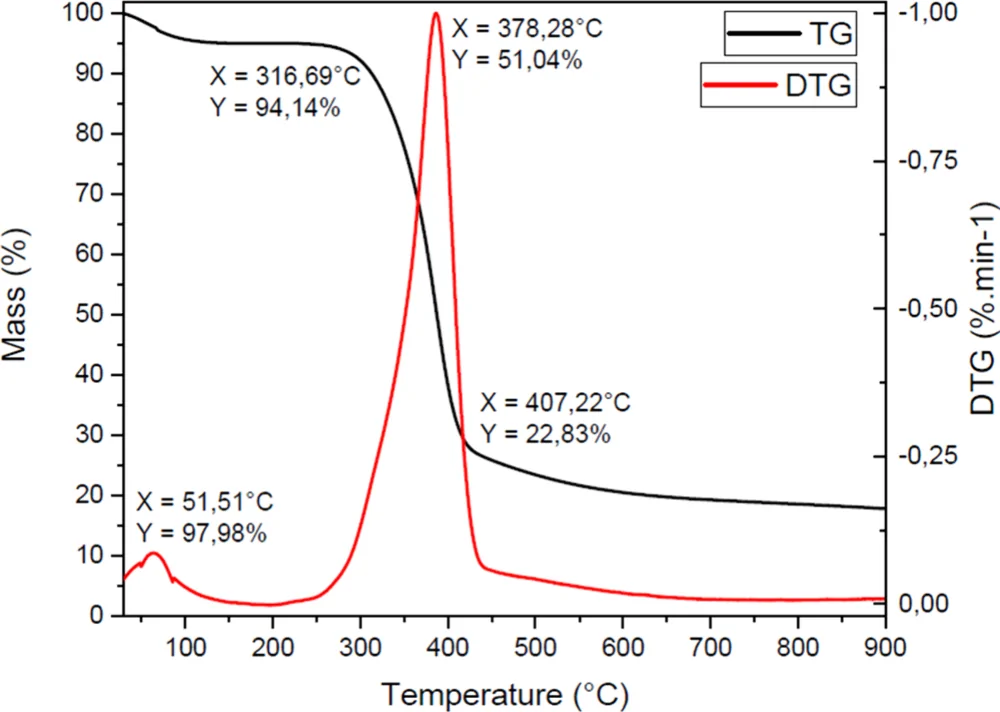
Thermogravimetric (TG) and derivative thermogravimetric (DTG) curves of sugarcane bagasse holocellulose.

**Figure 20:**
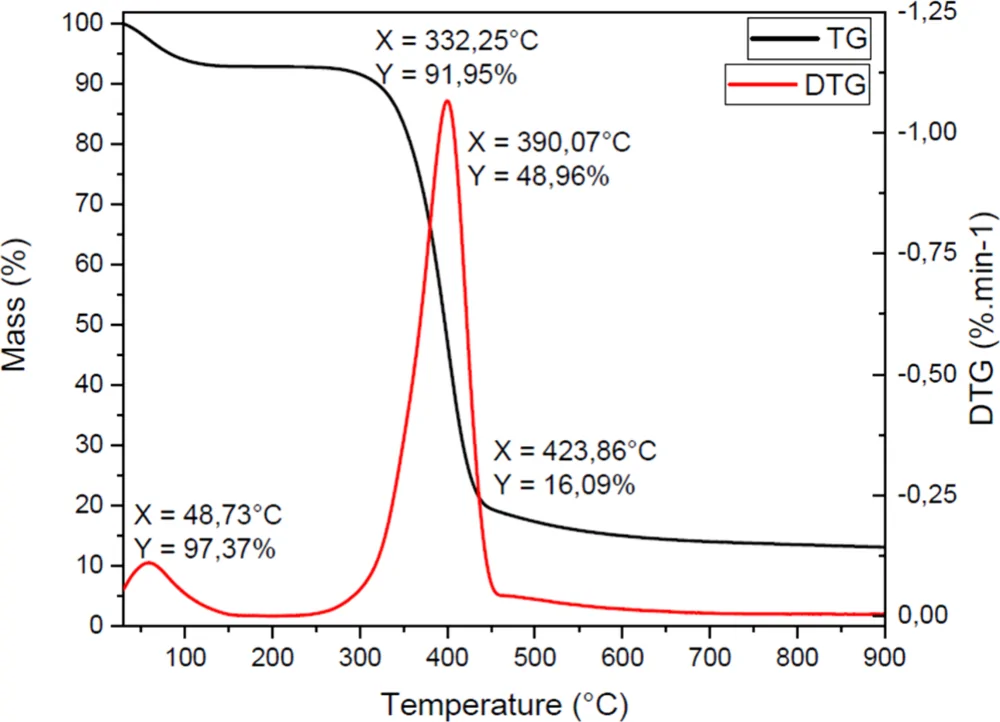
Thermogravimetric (TG) and derivative thermogravimetric (DTG) curves of sugarcane bagasse α-cellulose.

**Figure 21:**
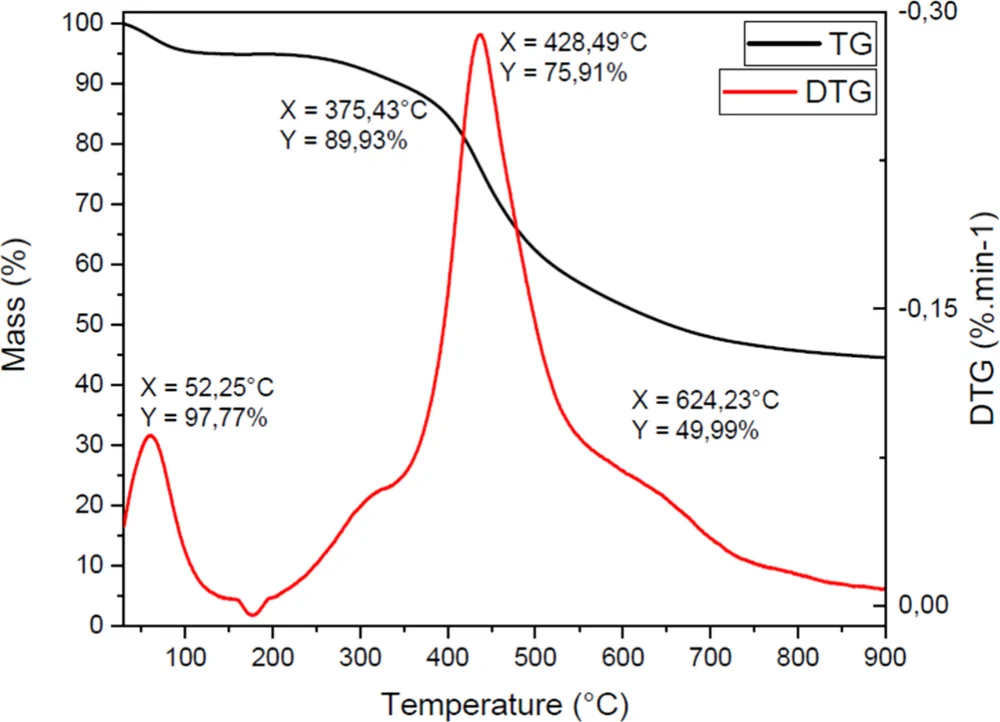
Thermogravimetric (TG) and derivative thermogravimetric (DTG) curves of bagasse lignin.

The comparison of these fractions provides a clearer understanding of the contribution of each lignocellulosic constituent to the thermal behavior of the native biomasses. As shown in Figures 16–21, for both eucalyptus and sugarcane bagasse, the transition from the extractive-free biomass to holocellulose resulted in a marked simplification of the DTG profile. The intermediate peak observed in the in natura and extractive-free biomasses disappeared after holocellulose isolation, indicating that thermally labile compounds associated with the removed fractions no longer contributed to the degradation process. As a result, the thermal behavior became predominantly controlled by the structural polysaccharides. The holocellulose profiles shown in Figures 16 and 19 exhibited the lowest *T*_max_ among the isolated fractions in both biomasses, reflecting the presence of hemicellulose, which is less thermally stable than cellulose and decomposes at lower temperatures.^47^

Although a distinct hemicellulose degradation peak was not resolved in either biomass, the DTG profiles of eucalyptus and sugarcane bagasse holocellulose (Figures 16 and 19, respectively) exhibited a broader base and a slight asymmetry toward lower temperatures compared with the corresponding α-cellulose fractions. This behavior suggests that hemicellulose degradation still overlaps with cellulose decomposition, producing a broadening of the DTG peak rather than an independent thermal event. Therefore, the absence of a well-defined hemicellulose peak should be interpreted not as the absence of hemicellulose degradation but rather as evidence of overlapping decomposition processes within the holocellulose fraction.

The α-cellulose fractions of eucalyptus and sugarcane bagasse exhibited very similar thermal behavior, characterized by a narrower degradation interval and a single, well-defined DTG peak. This behavior can be observed in the α-cellulose profiles presented in Figures 17 and 20. In both biomasses, the *T*_i_, *T*_onset_, *T*_max_, and *T*_offset_ values were higher than those observed for the corresponding holocellulose fractions, indicating greater thermal stability after hemicellulose removal. Since hemicellulose is the least thermally stable structural polysaccharide, its absence delays the onset of degradation and concentrates cellulose decomposition within a narrower temperature interval. The sharp and well-defined DTG peaks observed in Figures 17 and 20 are also consistent with the behavior previously observed for cotton and microcrystalline cellulose, reflecting the relatively homogeneous and partially crystalline structure of purified cellulose.

In contrast, the lignin fractions of eucalyptus and sugarcane bagasse exhibited the broadest TG and DTG profiles, characterized by multiple oscillations, a wide degradation interval, and comparatively low delta *Y* values. As shown in Figures 18 and 21, eucalyptus and sugarcane bagasse lignin, respectively, exhibited broad and irregular DTG profiles extending over a wide temperature range. Eucalyptus lignin presented a *T*_max_ of 420.84 °C and a delta *Y* of 52.34%, whereas sugarcane bagasse lignin reached a *T*_max_ of 428.49 °C with a delta *Y* of 55.58%. In both cases, mass loss continued above 500 °C before reaching stabilization, as observed in Figures 18 and 21, demonstrating considerably greater resistance to thermal degradation than the polysaccharide fractions. This behavior is consistent with previous studies on kraft and organosolv lignins and reflects the highly heterogeneous aromatic structure of lignin, composed of ether and carbon–carbon linkages with different bond dissociation energies, which promote gradual decomposition over a broad temperature range. Consequently, lignin contributes substantially to the thermal stability of lignocellulosic biomass and is primarily responsible for the broad degradation interval observed in the TG and DTG profiles of the native materials.

## 4 Conclusion

Thermal analyses enabled a clear understanding of the thermogravimetric behavior of biomass and its main structural components. All materials exhibited a DTG peak at 50–60 °C associated with moisture loss. Xylose showed low thermal resistance, with degradation occurring below 400 °C, while cellulose-based materials (cotton, microcrystalline cellulose, and eucalyptus and sugarcane bagasse α-cellulose) presented a narrow degradation range with a sharp *T*_max_ between 300 and 400 °C, characteristic of cellulose thermal transformation. Lignin (kraft, organosolv, eucalyptus, and sugarcane bagasse) exhibited broad decomposition ranges, higher *T*_max_ values, and greater thermal stability, consistent with previous reports in the literature.

For eucalyptus and sugarcane bagasse, TG/DTG profiles of in natura biomass and their structural fractions were similar, with progressive changes associated with the removal of extractives and hemicellulose. The in natura biomass showed moisture loss at ∼50 °C, *T*_onset_ at 312–350 °C, *T*_sh_ at 330–350 °C, *T*_max_ at 397–398 °C, and *T*_offset_ at 426–428 °C, corresponding to sequential degradation of extractives/hemicellulose, cellulose, and lignin. The gradual disappearance of the shoulder peak (*T*_sh_), together with the narrowing of the main DTG peak after sequential fractionation, demonstrated that the characteristic thermal profile of lignocellulosic biomass results from the overlap of degradation events associated with its structural constituents. Furthermore, DTG curve deconvolution quantitatively confirmed these overlapping thermal events, providing additional support for the interpretation of biomass thermal decomposition.

Unlike previous studies that generally evaluate individual biomasses or isolated fractions under different experimental conditions, this work systematically compares eucalyptus wood, sugarcane bagasse, and their structural fractions under standardized thermogravimetric conditions. This approach generated a unified comparative data set in which different biomasses and their main structural components were evaluated under the same experimental conditions, reducing the influence of methodological variability that often limits direct comparisons among studies. Consequently, the results provide a consistent experimental basis for comparing thermal parameters across different lignocellulosic materials and may contribute to improved reproducibility and a more reliable interpretation of TG/DTG data. The combination of sequential fractionation and DTG curve deconvolution provided a more robust interpretation of overlapping thermal events and established a direct relationship between the biomass composition and thermal behavior. Therefore, this study advances the interpretation of TG/DTG analyses of lignocellulosic biomass by providing a comparative and quantitative framework that contributes to a deeper understanding of pyrolysis mechanisms and supports the development of efficient, sustainable, and scalable bioenergy and biorefinery systems, promoting environmentally responsible biomass valorization technologies.

## Abbreviations

TG: thermogravimetry
DTG: derived from thermogravimetry
TV: volatile content (%)
*m*_i_: initial mass (g)
*m*_f_: final mass (g)
TC: ash content (%)
*m*: mass of the sample free of moisture (g)
*m*_1_: mass of the crucible (g)
*m*_2_: mass of the crucible plus the residue after the period in the muffle furnace (g)
TCF: fixed carbon content (%)
TC: ash content (%)
TV: volatile content (%)
ASTM: American Society for Testing and Materials
TAPPI: Technical Association of the Pulp and Paper Industry
*U*: moisture content (%)
Delta *Y*: mass variation (%)
*T*_i_: initial temperature
*m*_Ti_: mass at *T*_i_
*T*_onset_: onset temperature
*m*_Tonset_: mass at onset temperature
*T*_sh_: shoulder or deflection temperature
*m*_Tsh_: mass at shoulder temperature
*T*_max_: maximum degradation temperature
*m*_Tmax_: mass at maximum degradation temperature
*T*_offset_: offset temperature
*m*_Toffset_: mass at offset temperature
DSC: differential scanning calorimetry
